# SaVanT: a web-based tool for the sample-level visualization of molecular signatures in gene expression profiles

**DOI:** 10.1186/s12864-017-4167-7

**Published:** 2017-10-25

**Authors:** David Lopez, Dennis Montoya, Michael Ambrose, Larry Lam, Leah Briscoe, Claire Adams, Robert L. Modlin, Matteo Pellegrini

**Affiliations:** 1 Department of Molecular, Cell, and Developmental Biology, University of California, Los Angeles, CA 90095 USA; 2 Division of Dermatology, David Geffen School of Medicine, University of California, Los Angeles, CA 90095 USA; 3 Department of Microbiology, Immunology and Molecular Genetics, University of California, Los Angeles, CA 90095 USA

**Keywords:** Molecular signatures, Transcriptomic analysis, Tissue-specific expression, Heterogeneous samples, Visualization tools

## Abstract

**Background:**

Molecular signatures are collections of genes characteristic of a particular cell type, tissue, disease, or perturbation. Signatures can also be used to interpret expression profiles generated from heterogeneous samples. Large collections of gene signatures have been previously developed and catalogued in the MSigDB database. In addition, several consortia and large-scale projects have systematically profiled broad collections of purified primary cells, molecular perturbations of cell types, and tissues from specific diseases, and the specificity and breadth of these datasets can be leveraged to create additional molecular signatures. However, to date there are few tools that allow the visualization of individual signatures across large numbers of expression profiles. Signature visualization of individual samples allows, for example, the identification of patient subcategories a priori on the basis of well-defined molecular signatures.

**Result:**

Here, we generate and compile 10,985 signatures (636 newly-generated and 10,349 previously available from MSigDB) and provide a web-based Signature Visualization Tool (SaVanT; http://newpathways.mcdb.ucla.edu/savant), to visualize these signatures in user-generated expression data. We show that using SaVanT, immune activation signatures can distinguish patients with different types of acute infections (influenza A and bacterial pneumonia). Furthermore, SaVanT is able to identify the prominent signatures within each patient group, and identify the primary cell types underlying different leukemias (acute myeloid and acute lymphoblastic) and skin disorders.

**Conclusions:**

The development of SaVanT facilitates large-scale analysis of gene expression profiles on a patient-level basis to identify patient subphenotypes, or potential therapeutic target pathways.

**Electronic supplementary material:**

The online version of this article (10.1186/s12864-017-4167-7) contains supplementary material, which is available to authorized users.

## Background

Molecular signatures are collections of genes with an associated biological interpretation. For example, signatures can be generated from genes associated with specific cell types, diseases, or perturbations of cells by stimulatory signals. Signatures are typically generated from expression experiments that identify genes upregulated in a specific subset of samples when compared to a much broader group. Once generated, these signatures can be used to provide insights into the composition of heterogeneous samples. Signatures can also be composed of genes specifically associated with a disease. For example, molecular signatures from breast cancer samples have identified subphenotypes indistinguishable by traditional histological analyses [1], which can in turn be used to predict tumor invasiveness and inform patient treatment options.

Generally, the generation of molecular signatures involves the identification of a set of genes that are overexpressed in a subgroup of samples compared to the entire dataset. Several methods have been used to identify these genes, such as hierarchical clustering [2], machine learning [3], and neural networks [4]. In combination, these methods have led to the creation of thousands of molecular signatures and gene sets, which are compiled in established repositories such as MSigDB [5]. Furthermore, some signatures are manually curated for certain biochemically-determined pathways, such as REACTOME [6] and KEGG [7]. In general, the most popular current pathway enrichment tools, Ingenuity [8], GSEA [5], and DAVID [9], calculate enrichment of molecular signatures that have the highest statistical overlap with a gene list that the user has filtered by analysis of their expression study. By limiting the analysis to a single gene list, all of the individual variation of each expression profile is lost and further subcategorization of patient groups based upon these signatures is not immediately possible. Therefore the utility of signatures is limited by a lack of tools that are able to visualize the actual expression level of the signature genes within each user-supplied individual expression profile.

Furthermore, the current repositories of signatures are not exhaustive, and their signatures can be supplemented by additional signatures generated from large studies. For example, several consortia and large-scale projects have collected expression data with the aim of systematically profiling, and in some cases generating molecular signatures for a diverse group of cells, tissues, and diseases. These include collections of immune cell subsets [10–13], other primary and cultured cells [14, 15], tissue types [16, 17], cytokine-activated immune cells [18, 19], and skin diseases [20–22]. Collectively, these projects have produced over 3000 expression profiles for more than 600 cell and tissue types. The specificity and breadth of these expression experiments can be leveraged to create molecular signatures that are not currently represented in MSigDB that can then be used to interpret new datasets.

To overcome the limitations of existing tools, we have generated 636 new signatures from expression dataset collections and supplemented them with 10,349 signatures from MSigDB for a total of 10,985 signatures and have developed a web-based Signature Visualization Tool (SaVanT), to visualize these signatures in user-generated expression profiles. SaVanT is able to analyze user-supplied expression studies and visualize the average gene expression of molecular signatures across each individual expression profile. Through several examples, we show that SaVanT can be used to distinguish inflammatory patterns found between patients with different acute infections, identify the neoplastic cell type in leukemia samples, and provide insights into the immune response of several skin diseases. Through the visualization of molecular signatures, SaVanT allows users to efficiently leverage existing biological knowledge to interpret transcriptomic experiments.

### Implementation

SaVanT is a web-based tool that combines scripts implemented in Python and R. Python scripts process the user-submitted expression matrix and compute signature scores. R scripts perform ANOVA analyses and cluster the signature-sample matrix. After computation of the signature-sample matrix and clustering, Python scripts generate the HTML output and render the interactive heatmap. Visualization of the heatmap is provided by the HighCharts library (http://www.highcharts.com).

## Results

### Generation of new signatures

To leverage the vast number of reference expression profile repositories and add to MSigDB, we generated new molecular signatures using publicly-available expression data retrieved from a collection of repositories and sources (Table 1). Normalized data was used where available from the original study, but in lieu of preprocessed data, frozen robust multiarray analysis (fRMA) [23] normalization was used. Samples corresponding to biological replicates were averaged at the probe level, and genes with multiple probes were represented by the probe with the highest average intensity across all samples. In total, 4677 microarray profiles were retrieved to generate molecular signatures.

**Table 1:** Table 1

Expression Data Source	Reference	Platform	Normalization	# Signatures Generated
Human U133A/GNF1H Gene Atlas (BioGPS)	Su AI et al. (2004) *PNAS*	Affymetrix U133A/GNF1H	fRMA	84
Mouse MOE430 Gene Atlas (BioGPS)	Lattin JE et al. (2008) *Immunome Res.*	Affymetrix 430 2.0 Array	fRMA	94
Immunological Genome Project (ImmGen)	Heng TS et al. (2008) *Nature Immunology*	Affymetrix Gene 1.0 ST	Pre-processed	214
Human Cell Types (Swindell)	Swindell WR et al. (2013) *BMC Genomics*	Affymetrix Genome Plus 2.0	fRMA	24
Macrophage Activation	Xue J et al. (2014) *Immunity*	Illumina HumanHT-12 V3.0	Pre-processed	80
Primary Cell Atlas	Mabbott NA (2013) *BMC Genomics*	Affymetrix U133 Plus 2.0	fRMA	26
Skin Diseases (“DermDB”)	Inkeles MS et al. (2015) *J. Invest. Dermatol.*	Mixed	fRMA	23

Data sources for SaVanT signatures

Molecular signatures were generated from expression data by computing genome-wide ‘proportional median’ (PM) values. PM values are calculated by dividing the intensity of a microarray probe in a particular sample by the median intensity of the same probe across all samples in the corresponding data series. Therefore, high PM values are assigned to genes that are highly expressed in a certain sample relative to the others. A molecular signature consists of the top genes ranked in order of descending PM values. PM values have been previously used to generate signatures for a variety of skin diseases and conditions [20]. During the signature generation step, multiple samples for the same tissue/cell/disease are aggregated before PM computation. An average across these samples is computed for all genes, and from those averages, PMs values are computed by comparing to averaged samples of other tissues/cells/diseases. We note that the signatures we generated are ranked lists, while the signatures of MSigDB are unranked collections of genes. By default, we retain the top 50 PM-ranked genes to generate each molecular signature, but since we produce a value for all genes, the signatures can also be generated using a variable number of genes. Using this PM metric, 636 ranked molecular signatures were created. The signatures represent a diverse set of biological states, as a consequence of the variety of sources used: we generated signatures for 158 tissue types, 277 cell types, 70 primary cells, 114 molecular perturbations, and 17 skin diseases.

To assess and validate the use of proportional median values to create molecular signatures, we have annotated the genes from two representative signatures generated from the Human Primary Cell Atlas: an adipocyte-specific signature and a keratinocyte-specific signature (Additional file 1: Table S1 and Additional file 2: Table S2). The annotations assigned to the top genes (by PM rank) are characteristic of the distinct biology underlying the samples. For example, the adipocyte signature contains genes required for fatty acid processing and metabolism (fatty acid binding protein 4 [FABP4]), lipogenic proteins (lipogenic protein 1/THRSP), regulatory genes (adipogenesis regulatory factor/C10orf116), as well as genes known to be uniquely expressed in adipocytes, such as adiponectin (ADIPOQ). Similarly, the keratinocyte signature contains several keratin genes (keratin 6AII, keratin 14I, keratin 2II), envelope proteins (small proline-rich protein 1A [SPRR1A]), and regulatory genes involved in keratinocyte differentiation and maintenance (keratinocyte differentiation-associated protein [KRTDAP]). The enrichment of adipocyte- and keratinocyte-related annotations for the top genes (by PM rank) in each respective signature suggests that our PM values capture genes that are specifically representative of the cell type or state of interest.

### Visualization of molecular signatures

**Fig. 1:**
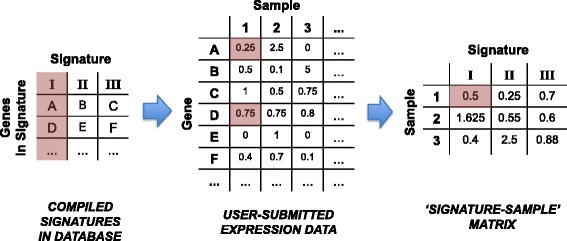
Constructing ‘Signature-Sample’ Matrix From Expression Data. The SaVanT pipeline converts user-submitted expression data into a signature-sample matrix whose columns are the submitted samples and rows are the user-selected molecular signatures. By default (shown above), every cell in this matrix contains the average value of signature genes for a particular signature-sample combination. The breakdown for an example cell in the signature-sample matrix is shown in red. The matrix value is computed by looking up the genes in any given user-selected signature in the SaVanT database (middle panel) and subsequently averaging the values of these genes in a particular sample in the user-submitted data (left and right panels). Above, samples are designated with numbers, genes with letters, and signatures with Roman numerals

In order to visualize molecular signatures across any expression data of interest, we have developed the Signature Visualization Tool (SaVanT). SaVanT is a web-accessible tool that accepts matrices of gene expression data (i.e., from RNA-seq or microarray experiments) and produces a visual representation of the signatures across the submitted samples as an interactive heatmap. The key step in the SaVanT pipeline is to create a ‘sample-signature’ matrix whose columns are the input samples and the rows are the user-selected molecular signatures (Fig. 1). In order to create this matrix, SaVanT accepts as input a matrix of gene expression values (gene symbols as rows and sample names as columns). A specification and example of expected input are provided on the main page of SaVanT. Using the default settings, every cell in this matrix contains the average value of signature genes for a particular signature-sample combination. This average value is computed by looking up the top genes for the user-selected signature in the SaVanT database and subsequently averaging the values of these genes in a particular sample in the user-submitted data. The default in SaVanT is to use the top 50 genes (by PM value) in each signature, but we also allow the size of signatures to be changed to include the top 10, 25, 100, 250, 500 or 1000 genes. The sample-signature matrix is displayed by SaVanT as an interactive heatmap that can be optionally clustered along its axes. Alternatively, the ‘sample-signature’ matrix can consist of sums instead of mean values, and can be converted to z-scores or filtered by minimum values.

**Fig. 2:**
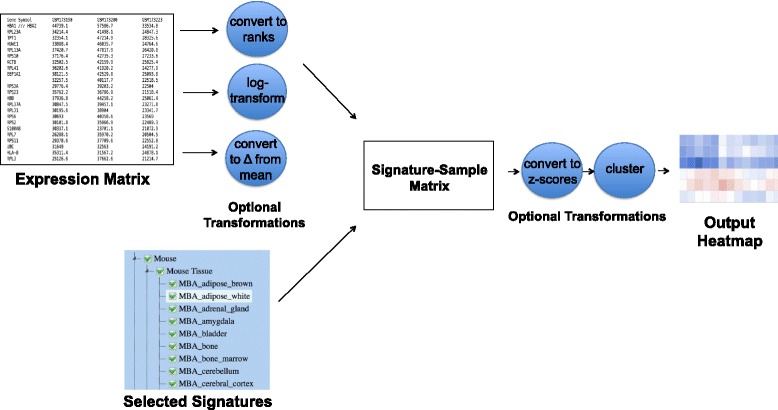
SaVanT Pipeline. In the first step, an expression matrix containing values for genes in several samples is optionally converted to ranked lists of genes in samples or log-transformed. The expression matrix is then converted into a signature-sample matrix as described in Fig. 1 using the selected signatures. Optionally, the signature-sample matrix is converted to differences from mean values, converted to z-scores, and/or clustered to produce a final heatmap

In order to enhance the visualization of the ‘sample-signature’ matrix, several optional steps can be used to transform the user-uploaded data or the ‘sample-signature’ matrix (Fig. 2). For example, to dampen the effects of the large dynamic ranges characteristic of RNA-seq data, expression values can be log-transformed, converted to ranks, as well as shown as the difference from the mean value of all the samples. For optimal results, uploaded datasets should be also preprocessed to filter out transcripts or probes near technical detection limits (e.g., probes with low intensities or transcripts with low RPKMs). Once the sample-signature matrix is computed, its values can be converted to z-scores. On the submission page, an interactive description of the steps to create the matrix is shown, reflecting the chosen parameters. Clustering of the sample-signature matrix can be performed using several distance metrics (Euclidean distance or Pearson correlation) as well as different linkage parameters. The heatmap produced by SaVanT is interactive, and additional information (such as the sample-signature combination, *p*-values, and the matrix value) are shown as hover-over boxes.

Statistical significance of a signature score can be shown within SaVanT by selecting the option to compute a null distribution under “Statistical Options”. This feature computes p-values by permuting the gene expression data and analyzing the distribution of genes in signatures relative to the distribution of randomly selected genes. By default, 10,000 permutations are performed, although the user can select a different number. The p-values are shown for each score when the mouse hovers over the heatmap, but the p-values themselves can be visualized on the heatmap by selecting the option under “Statistical Options”. In order to account for signature size, signature scores can also be scaled by the square root of the number of signature genes by selecting “scale signature values by the square root of signature genes” under “Display Options”. Finally, there is also an option to remove signatures with less than a user-selectable minimum number of genes.

### Analyses of example datasets

To demonstrate the capabilities of SaVanT, we provide biologically-motivated examples using publicly-available datasets retrieved from GEO [24].

#### Cell type identification within tissue samples

**Fig. 3:**
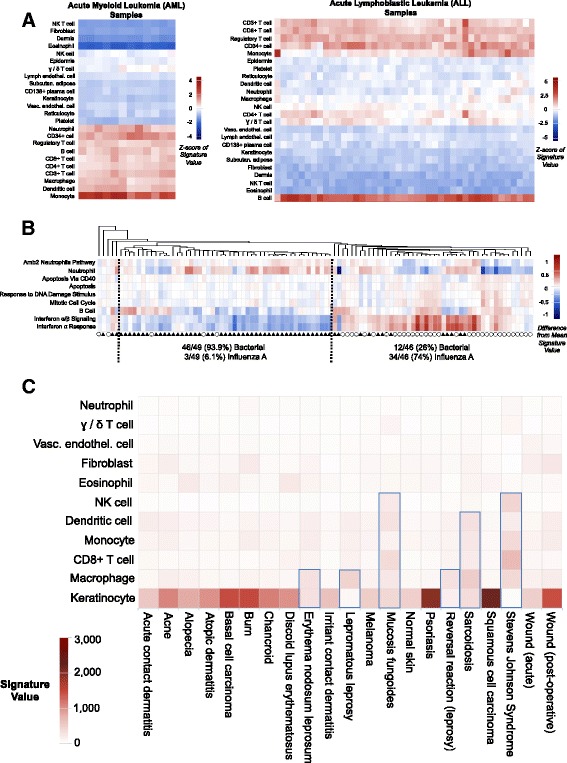
SaVanT Distinguishes Between Patients, Cell Types, and Underlying Biology. **a** SaVanT output for expression data from acute myeloid leukemia (AML) and acute lymphoblastic leukemia (ALL) patients. ‘Signature value’ refers to the average of gene expression values in a signature. Z-scores (across the entire signature value matrix) are shown for both heatmaps. **b** SaVanT output for expression data from 99 patients with acute infections (either Influenza A or bacterial pneumonia). The infection type for each patient is represented by a hatched circle (Influenza A) or filled triangle (bacterial pneumonia). The numbers below each cluster quantify the proportion of infection types. The difference between the signature values and the average signature value per signature is shown. **c** SaVanT output for expression data from different skin diseases

SaVanT can be used to identify the relative abundance of cell types found within tissue samples. To demonstrate this capability, we retrieved samples from patients with acute myeloid leukemia (AML) (GEO accession GSE29883) and acute lymphoblastic leukemia (ALL) (GEO accession GSE32962) (Fig. 3A). Using a panel of signatures representing different hematopoietic cells, SaVanT produced heatmaps identifying the principal cell type in AML samples (monocytes) and ALL samples (B cells). Furthermore, the heatmap identifies one ALL sample that may be misclassified (the first sample in the heatmap), although we could not find metadata to support this.

#### Discrimination of disease phenotypes

Often the main goals of expression studies of clinical samples are to distinguish between clinical phenotypes and to identify the molecular signatures that differ between phenotypes to provide insight on disease pathogenesis. If the group structure of the submitted samples is known, the tag ‘SAVANT_GROUP’ can be included in the submitted expression matrix with integers designating group membership of the samples, which automatically runs an ANOVA analysis on the signature-sample matrix (Additional file 3: Figure S1). A detailed description of the necessary tags required to enable ANOVA analysis is described in the input specification accessible from the main page of SaVanT. To demonstrate this feature of SaVanT, expression data was retrieved from a study profiling expression of whole blood samples collected daily from 17 patients with either influenza A or bacterial pneumonia [26]. The study found enrichment of interferon, cell cycle genes, apoptosis, DNA damage, B cell, CD4+ T helper cells, and neutrophils in influenza-induced versus bacterial pneumonia (i.e., upregulation in viral samples). We used SaVanT to visualize equivalent gene signatures from MSigDB or cell type-specific expression profiles on a per-patient basis, filtering for those signatures that are statistically different between bacterial pneumonia and influenza. To accomplish this, we provided the type of sample (pneumonia or influenza) as the ‘SAVANT_GROUP’ to trigger an ANOVA analysis, and retained the signatures with the lowest *p*-values. Additionally, other signatures such as apoptosis and DNA damage were included as negative controls. The clustered heatmap produced by SaVanT separates the acute infection samples into two groups: the predominantly influenza cluster was characterized by higher signature values for type I interferon pathways, B cells, cell-cycle, DNA damage, and apoptosis (Fig. 3B). The bacterial pneumonia cluster was composed of 92% bacterial pneumonia samples, characterized by higher neutrophil signature values relative to influenza. Five other samples were clustered as outliers. In addition to identifying the main clusters between disease groups, the SaVanT analysis displays intra-disease differences in molecular and cellular pathways. For example, there are two different bacterial subclusters in which one group has a higher B cell signature while the other has a higher neutrophil signature. Furthermore, upon examination of the influenza group we can see that the misclassified bacterial pneumonia samples still have higher neutrophil signatures, but also have high type 1 interferon signatures, potentially identifying the reason for misclassification and targeting for further investigation.

#### Dermatoses

Lastly, in order to illustrate the analyses of heterogenous tissue samples, we used expression data from a collection of skin diseases [20] and analyzed these using signatures for specific cell types found in the skin (Fig. 3C). The predominant signature for most samples is that of keratinocytes, which illustrates that while our signature values cannot be interpreted as quantitative estimates of cell type fractions, a higher relative value does reflect that the underlying cell type is more abundant than those associated with other lower scoring signatures. Within these dermatoses we also find several samples that have weaker keratinocyte signatures, but higher values for other signatures (designated by blue boxes). For example, the macrophage signature is elevated in leprosy lesions (erythema nodosum leprosum, lepromatous leprosy, and reversal reaction), as would be expected from the presence of macrophages within the granulomas in these biopsies. Furthermore, signatures derived from hematopoietic cells are elevated in tissue samples from patients with Stevens Johnsons disease, which are collected from blister fluid, along with mucosis fungoides, a T cell neoplasm, and sarcoidosis, which also typically has abundant granulomas. Overall, these signatures help interpret the components of these skin biopsies, which may in large part underlie the differences in gene expression between them.

## Discussion

SaVanT provides an interactive platform for compiling and visualizing molecular signatures in order to interpret user-submitted data. The newly-generated signatures that supplement MSigDB, leverage the specificity and depth of large expression studies to capture the biology pertaining to specific diseases, cell types, and immune states. The functions of the top genes in our signatures often reflect signature-specific characteristics. The adipocyte and keratinocyte signatures serve as representative examples, with each of their top 10 genes reflecting specific association to the differentiated cell lineages.

Moreover, in addition to compiling a large database of signatures, we also provide a framework that enables their mining and visualization to help interpret user-supplied expression data. To this end, we provide very flexible options to enable different analyses. For example, signature values can be filtered to only display those above a certain threshold or clustered using a number of different options. Furthermore, SaVanT has been optimized to quickly scan more than 10,000 signatures in seconds, thus allowing all signatures to be computed against user-uploaded expression matrices.

The power of SaVanT is illustrated in the examples shown in Fig. 3. The fundamental objectives of each analysis are distinct: identification of inflammatory states that differentiate two clinical presentations (Fig. 3A), identification of the neoplastic cell types in a liquid tumor (Fig. 3B), and gaining insights into the compositions of heterogeneous biopsies (Fig. 3C). Viral infection, including influenza is characterized by a strong induction of a type I interferon antiviral response, composed of genes induced by interferon alpha and beta [25, 26], and this is reflected in the signature-sample heatmap. The neoplastic cell types present in a leukemia can be seen using the signature-sample heatmap, and misclassified patients identified. Lastly, the cell type compositions of skin biopsies from a number of dermatoses can be determined from expression data. In the future we plan to continue to develop SaVanT by adding more signatures, along with additional features that facilitate the interpretation of complex expression patterns.

SaVanT complements existing tools that analyze transcriptomic datasets using gene signatures, such as those inferring cellular composition [27]. Although several tools exist to identify or analyze enrichment of gene sets and signatures within gene expression data, we believe SaVanT distinguishes itself by its comprehensive set of signatures, an intuitive web-accessible interface, interactive output, and rapid runtime. Table 2 provides a comparison to other tools (GSEA [5], BubbleGUM [28], GSVA [29], PLAGE [30], and ssGSEA [31]). Although many of these tools use the MSigDB repository as the source of their gene sets, we also provide an additional 636 newly-generated signatures that describe cell types, tissue-specific expression, and disease states. Furthermore, many other tools require processing an expression matrix into a specific format, as well as downloading and running an R package or Java archive that requires heavier bioinformatics expertise and experience. Our tool is especially useful even when phenotype data is not readily available or when the objective is a global (i.e., not pairwise) comparison between samples. We believe the combination of these differences distinguishes SaVanT from other existing tools.

**Table 2:** **Table 2**

Tool/Resource	Analysis Objective	Number of Signatures or Datasets	Number of Samples Analyzed	Input	Output	Interface and Requirements	Runtime
SaVanT	Visualization of molecular signatures across samples	10,985 signatures	1–150 samples	Gene expression matrix (gene symbols and values)	Interactive heatmap	Website/Browser	75 s (50 samples, 25,219 genes, 4729 signatures, with ANOVA)
GSEA	Identification of significant or differential gene sets and signatures	User-defined; MSigDB supported (up to 18,026 gene sets)	Two or more biological states (with replicates)	Expression dataset and phenotype data	Enrichment plots and lists	Java Archive Download	4 min (50 samples, 4729 signatures, 9096 genes, 1000 permutations)
BubbleGUM	Extraction and visualization of molecular signatures and gene sets	User-defined; MSigDB supported (up to 18,026 gene sets)	2+ samples	GCT file (expression dataset) and phenotype data	Graphical plots	Java Archive Download	5 min (13 samples, 75 signatures, 1000 permutations)
GSVA	Estimation of variation in pathway and signature genes across samples	User-defined; MSigDB supported (up to 18,026 gene sets)	2+ samples	Gene expression matrix and gene set data	Score matrix of enrichment scores	R package (Bioconductor)	3 min (30 samples, 100 gene sets, 20,000 genes)
PLAGE	Quantification of pathway activity across samples	400 pathways from KEGG	2+ samples	Gene expression matrix	Heatmap of pathway activity levels	Website/Browser	N/A (Could not access website)
ssGSEA	Determine enrichment of a gene set within dataset	User-defined; MSigDB supported (up to 18,026 gene sets)	2+ samples	GCT file with expression estimates	Matrix of enrichment projections	R package (or via browser using GenePattern)	2 min (50 samples, 326 gene sets, 10,100 genes)

Previously published tools for the analysis of gene signatures

## Conclusions

Together with the generation of 636 new molecular signatures, we have compiled them with 10,349 previously available from MSigDB. These signatures capture cell-specific, tissue-specific, and perturbation-derived information that can be leveraged to extract biological interpretation from newly-generated expression profiles. In order to leverage these signatures and to facilitate the large-scale analysis of gene expression profiles on a patient-level basis, we have developed a web-based visualization tool, SaVanT, and demonstrated its ability to distinguishes between patients, cell types, and underlying biology in a variety of publicly-available datasets.

## Availability and requirements

SaVanT is implemented as a web-accessible resource, available at http://newpathways.mcdb.ucla.edu/savant-dev/.

Project Name: Signature Visualization Tool (SaVanT).

Project Home Page: http://newpathways.mcdb.ucla.edu/savant-dev/

Operating system(s): Platform independent.

License: GNU General Public License.

## Methods

### Expression data retrieval and processing

Microarray data was retrieved from Gene Expression Omnibus (GEO) for samples and series listed in Supplementary Table 2. Raw CEL files were processed using the ‘affy’ R package and were normalized using the ‘frma’ R package in conjunction with the respective ‘frmavecs’ package for the platform used. Intensities for multiprobe genes were taken from the probe with the highest mean expression across all samples. Samples annotated as biological replicates in the GEO series description were combined by taking the average value of probes in the replicates.

### Signature generation

‘Proportional median’ (PM) values were calculated by dividing the intensity of a probe in a particular sample by its median value across all samples. For PM calculations, datasets from different sources were considered independently (i.e., the denominator was composed of only samples within a certain series when calculating PMs for a sample within that series). PMs were calculated at the probe level, and PM values were subsequently associated with gene symbols using the platform-specific annotation tables from GEO.

### Signature visualization

In order to produce the sample-signature heatmap with SaVanT, the user-submitted expression matrix is processed by a series of scripts. Ambiguous values, such as those for gene symbols appearing multiple times in the user input, are resolved by taking the average value of all instances. Optional transformations (log-transformation and/or conversion to ranks, in that order) are performed on the input expression matrix, and the ‘sample-signature matrix’ is created by taking the average (or sum, optionally) of expression values for genes in every signature-sample pair. If conversion to z-scores is selected, the mean and standard deviation is computed for the entire ‘sample-signature’ matrix, which are used to convert the values to z-scores. Clustering is optionally performed by the R ‘heatmaps.2’ function of the ‘gplots’ package. The signature-sample matrix is displayed interactively using a modified version of the HighCharts JavaScript library.

### Example datasets

The example datasets use publicly available data of AML samples (GEO accession GSE29883), ALL samples (GEO accession GSE32962), and acute infection patients ([26]). Default settings were used, except as follows: for the analysis of AML and ALL samples, samples were log-transformed and normalizated to z-scores; and for the analysis of acute infections, values were transformed to an average difference from mean.

## Additional files

Additional file 1: Signature genes for adipocytes. (DOCX 14 kb)

Additional file 2: Signature genes for keratinocytes. (DOCX 13 kb)

Additional file 3: Figure S1. SaVanT performs ANOVA analysis on samples with known group memberships. For samples where group memberships are known a priori, a ‘SAVANT_GROUP’ row can be added to the gene expression matrix to perform an ANOVA analysis within SaVanT. An example result of an ANOVA analysis is shown. Signatures were filtered for those that are significant (*p*-value <0.0001). The asterisks in the rightmost column indicate the significance level for each signature: * < = 0.01; ** < = 0.001; *** < = 0.0001 (PDF 693 kb)

## Abbreviations

ADIPOQ: Adiponectin
ALL: Acute lymphoblastic leukemia
AML: Acute myeloid leukemia
FABP4: Fatty acid binding protein 4
fRMA: Frozen robust multiarray analysis
GEO: Gene Expression Omnibus
KEGG: Kyoto Encyclopedia of Genes and Genomes
KRTDAP: Keratinocyte differentiation-associated protein
PM: Proportional median
SPRR1A: small proline-rich protein 1A
