# Supplementary material for: SaVanT: a web-based tool for the sample-level visualization of molecular signatures in gene expression profiles
Source: BMC Genomics. 2017 Oct 25;18:824. doi: 10.1186/s12864-017-4167-7 (PMC5657101; doi:10.1186/s12864-017-4167-7)
Supplement: Supplementary file 1 — Signature genes for adipocytes. (DOCX 14 kb) [file 12864_2017_4167_MOESM1_ESM.docx]

**Table S1**

| **Gene Symbol** | **PM Value** | **Gene Description** | **Notes** |
| --- | --- | --- | --- |
| *FABP4* | 577.23 | Fatty Acid Binding Protein 4, Adipocyte | encodes the fatty acid binding protein found in adipocytes; FABPs roles include fatty acid uptake, transport, and metabolism. |
| *ADIPOQ* | 563.24 | Adiponectin, C1Q And Collagen Domain Containing | gene is expressed in adipose tissue exclusively; encoded protein circulates in the plasma and is involved with metabolic and hormonal processes |
| *COL3A1* | 519.97 | Collagen, Type III, Alpha 1 | encodes the pro-alpha1 chains of type III collagen, a fibrillar collagen that is found in extensible connective tissues such as skin, lung, uterus, intestine and the vascular system |
| *ADH1B* | 494.47 | Alcohol Dehydrogenase 1B (Class I), Beta Polypeptide | a member of the alcohol dehydrogenase family; metabolize a wide variety of substrates, including ethanol, retinol, other aliphatic alcohols, hydroxysteroids, and lipid peroxidation products |
| *THRSP* | 468.30 | Thyroid Hormone Responsive; Lipogenic Protein 1 | shown to be expressed in liver and adipocytes, particularly in lipomatous modules ; also found to be expressed in lipogenic breast cancers |
| *RBP4* | 458.86 | Retinol Binding Protein 4, Plasma |  |
| *COL1A2* | 452.31 | Collagen, Type I, Alpha 2 |  |
| *SRPX* | 350.80 | Sushi-Repeat Containing Protein, X-Linked |  |
| *TIMP4* | 338.46 | TIMP Metallopeptidase Inhibitor 4 |  |
| *C10orf116* | 317.78 | Adipogenesis Regulatory Factor |  |
