# Supplementary material for: SaVanT: a web-based tool for the sample-level visualization of molecular signatures in gene expression profiles
Source: BMC Genomics. 2017 Oct 25;18:824. doi: 10.1186/s12864-017-4167-7 (PMC5657101; doi:10.1186/s12864-017-4167-7)
Supplement: Supplementary file 2 — Signature genes for keratinocytes. (DOCX 13 kb) [file 12864_2017_4167_MOESM2_ESM.docx]

**Table S2**

| **Gene Symbol** | **PM Value** | **Gene Description** | **Notes** |
| --- | --- | --- | --- |
| *SPRR1A* | 1238.97 | Small Proline-Rich Protein 1A | cross-linked envelope protein of keratinocytes; first appears in the cell cytosol, but ultimately becomes cross-linked to membrane proteins by transglutaminase |
| *KRTDAP* | 1186.22 | Keratinocyte Differentiation-Associated Protein | may function in the regulation of keratinocyte differentiation and maintenance of stratified epithelia |
| *KRT6A* | 1175.16 | Keratin 6A, Type II | consist of basic or neutral proteins which are arranged in pairs of heterotypic keratin chains coexpressed during differentiation of simple and stratified epithelial tissues |
| *KRT14* | 1173.01 | Keratin 14, Type I | usually found as a heterotetramer with two keratin 5 molecules, a type II keratin; together they form the cytoskeleton of epithelial cells. |
| *SPRR1B* | 1040.41 | Small Proline-Rich Protein 1B | cross-linked envelope protein of keratinocytes; diseases associated with SPRR1B include epidermolytic hyperkeratosis |
| *FLG* | 883.49 | Filaggrin |  |
| *FLG2* | 880.08 | Filaggrin Family Member 2 |  |
| *DSC1* | 772.33 | Desmocollin 1 |  |
| *KRT2* | 678.45 | Keratin 2, Type II |  |
| *DMKN* | 647.12 | Dermokine |  |
