# Supplementary figures and images for: SaVanT: a web-based tool for the sample-level visualization of molecular signatures in gene expression profiles
Source: BMC Genomics. 2017 Oct 25;18:824. doi: 10.1186/s12864-017-4167-7 (PMC5657101; doi:10.1186/s12864-017-4167-7)

Values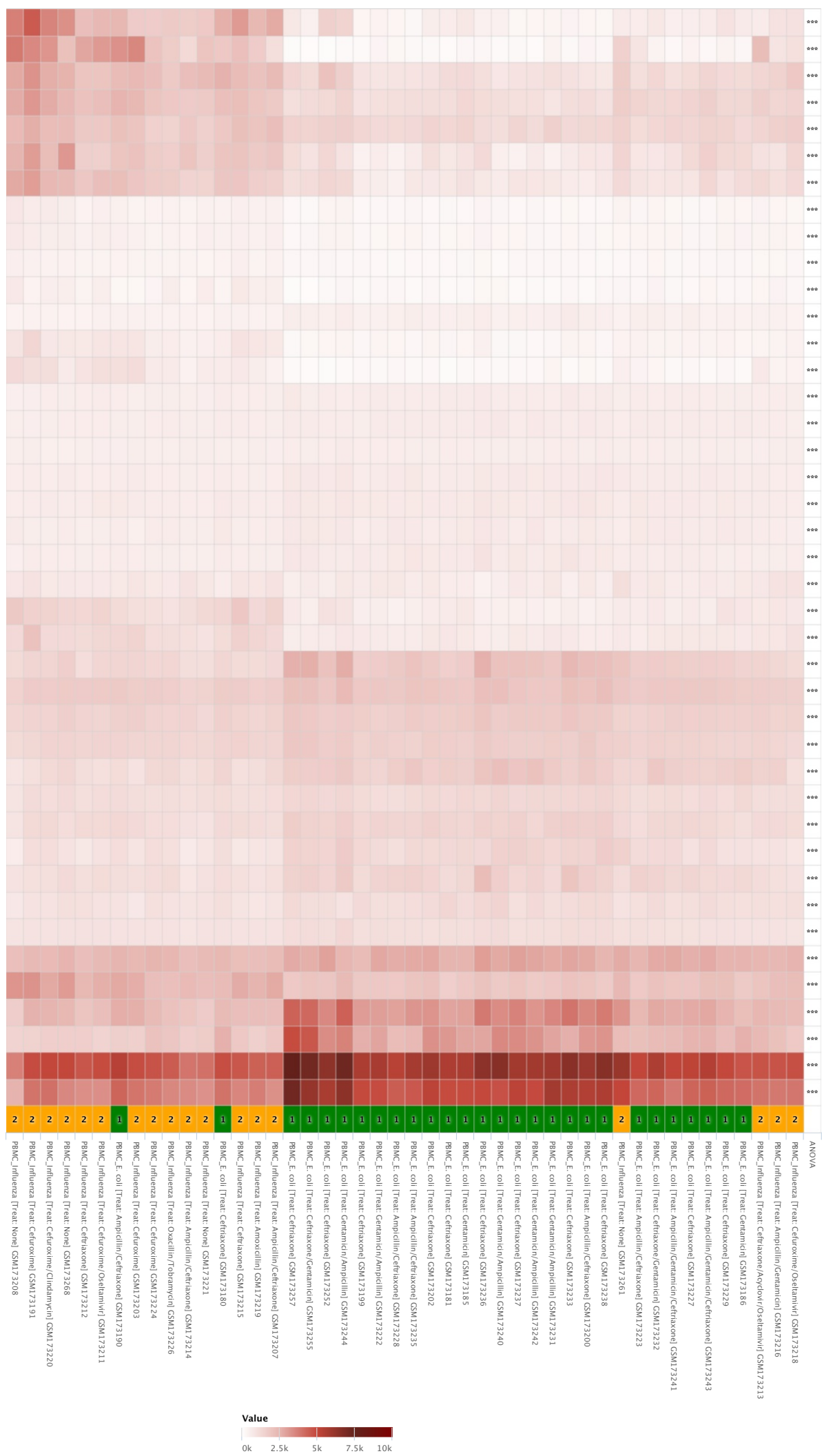

ANOVA

Supplement: Supplementary file 3 — SaVanT performs ANOVA analysis on samples with known group memberships. For samples where group memberships are known a priori, a ‘SAVANT_GROUP’ row can be added to the gene expression matrix to perform an ANOVA analysis within SaVanT. An example result of an ANOVA analysis is shown. Signatures were filtered for those that are significant (p-value <0.0001). The asterisks in the rightmost column indicate the significance level for each signature: * < = 0.01; ** < = 0.001; *** < = 0.0001 (PDF 693 kb) [file 12864_2017_4167_MOESM3_ESM.pdf]
